# The various role of microRNAs in breast cancer angiogenesis, with a special focus on novel miRNA-based delivery strategies

**DOI:** 10.1186/s12935-022-02837-y

**Published:** 2023-02-10

**Authors:** Min Yang, Ying Zhang, Min Li, Xinglong Liu, Mohammad Darvishi

**Affiliations:** 1 College of Traditional Chinese Medicine, Jilin Agricultural Science and Technology University, Jilin, 132101 China; 2 Infectious Diseases and Tropical Medicine Research Center (IDTMRC), Department of Aerospace and Subaquatic Medicine, AJA University of Medical Sciences, Tehran, Iran

**Keywords:** microRNAs, Breast cancer angiogenesis, angiomiRs, microRNA-based therapy

## Abstract

After skin malignancy, breast cancer is the most widely recognized cancer detected in women in the United States. Breast cancer (BCa) can happen in all kinds of people, but it's much more common in women. One in four cases of cancer and one in six deaths due to cancer are related to breast cancer. Angiogenesis is an essential factor in the growth of tumors and metastases in various malignancies. An expanded level of angiogenesis is related to diminished endurance in BCa patients. This function assumes a fundamental part inside the human body, from the beginning phases of life to dangerous malignancy. Various factors, referred to as angiogenic factors, work to make a new capillary. Expanding proof demonstrates that angiogenesis is managed by microRNAs (miRNAs), which are small non-coding RNA with 19–25 nucleotides. MiRNA is a post-transcriptional regulator of gene expression that controls many critical biological processes. Endothelial miRNAs, referred to as angiomiRs, are probably concerned with tumor improvement and angiogenesis via regulation of pro-and anti-angiogenic factors. In this article, we reviewed therapeutic functions of miRNAs in BCa angiogenesis, several novel delivery carriers for miRNA-based therapeutics, as well as CRISPR/Cas9 as a targeted therapy in breast cancer.

## Introduction

In women all over the world, the most common cancer site is the breast [1]. Breast cancer (BCa) is diagnosed in 2.1 million women per year. Early diagnosis is critical for improving patient survival since it reveals the best treatment approach for each case [2–4]. In the United States, about 10,000 40-year-old women are diagnosed with invasive BCa. The Asian BCa Association reported that 13% of the women diagnosed with BCa were 40 years old, and 5% were 35 years old [5, 6]. BCa is considered more common as people get older, due to the accumulation of somatic mutations in the mammary glands [7, 8]. In 2018, approximately 266,120 women will be detected with invasive BCa in the United States, while 63,960 will be detected with in situ BCa. Invasive BCa will affect about 2550 males in 2018. Breast cancer affects about one in every 1000 males at some point in their lives [9, 10]. BCa may be classified into three classes based on molecular and histological evidence: BCa that expresses hormone receptors (estrogen receptor (ER +) or progesterone receptor (PR +), BCa that expresses human epidermal receptor 2 (HER2 +), and Triple-negative breast cancer or TNBC (ER, PR, HER2) [11].

Angiogenesis is undoubtedly the system of making capillaries through pre-current ones [12]. Angiogenesis is a critical mechanism involved in a variety of pathological and physiological processes. This is a highly controlled process in physiological settings. Diabetic retinopathy, Psoriasis, and cancer are all examples of pathological angiogenesis. Angiogenesis is necessary for appropriate nutrition and the elimination of metabolic wastes from tumor locations during tumor development [13, 14]. It has commonly assumed that pre-cancerous tissues develop angiogenic abilities as they progress into cancer [15]. Angiogenesis in tumors is controlled by a variety of cytokines and genetic factors [16]. Angiogenesis occurs as a fibrin clot forms on an established blood vessel's adventitial surface and new capillaries develop after that. Vascular permeability increases, and the vessel wall deteriorates locally (i.e., extracellular matrix (ECM) or basement membrane), marking the initial step's start. Endothelial cells join the stroma of the tumor, migrate against a signal like vascular endothelial growth factor (VEGF) or fibroblast growth factor (FGF), and they proliferate behind the cutting hand [17, 18]. Since they lack defense from other cell types, the cells may be more vulnerable to agents that interfere with their proliferation at this time [19, 20]. Pericytes are recruited next, followed by smooth muscle cells, in the process of vessel development [21].

**Fig. 1:**
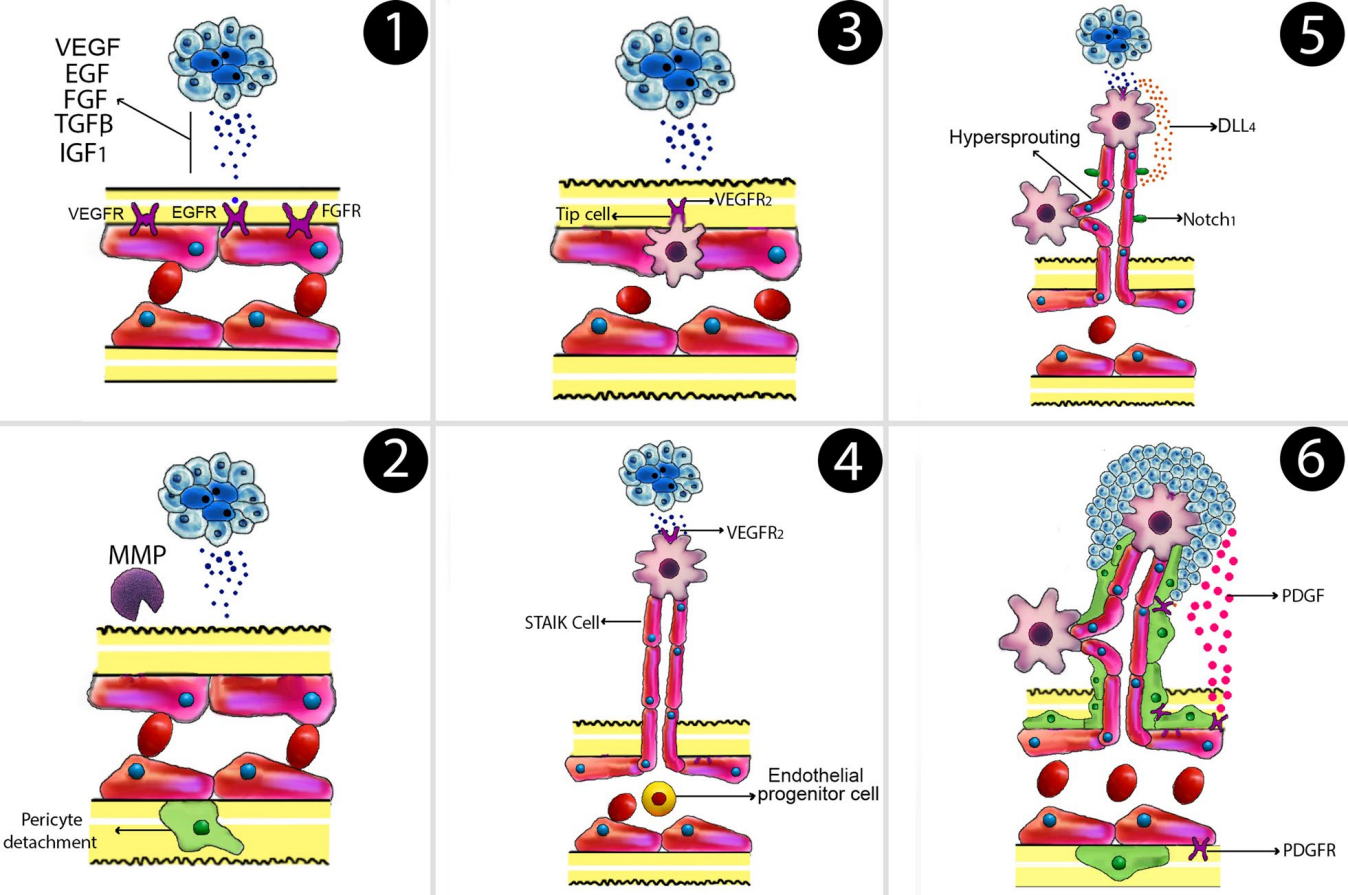
Angiogenesis HIF-1, which leads to the secretion of pro-angiogenic factors, now the most significant of which being VEGF. Hypoxia also causes basement membrane disintegration and pericyte datachment by increasing protease expression. Angiogenic factor gradients attract specialized endothelial cells. Endothelial cells are transformed into strongly proliferative stalk cells that form the new vessel’s main body. DLL4 production is stimulated by VEGF, that interacts to Notch-1 receptors and suppresses Vascular Endothelial Growth Factor receptor (VEGFR). Pericyte attachment is stimulated by PDGF, which lowers proliferation and sensitivity to VEGF. Tumor growth is aided by the availability of blood. *EGF* epidermal growth factor, *FGF* fibroblast growth factors, *TGF-β* transforming growth factor beta, *HIF-1* hypoxia activates hypoxia-inducible factor 1; VEGF: vascular endothelial growth factor, *DLL4* delta-like 4, *PDGF* platelet-derived growth factor, *IGF1* insulin-like growth factor 1

MicroRNAs (miRNAs) are short non-coding RNAs (ncRNAs) of 21–25 nucleotides [22]. MiRNAs regulate more than 30 percent of all gene expression in vivo [23, 24]. More than 1000 human miRNAs have been discovered, each with the ability to regulate hundreds of genes [25]. In 1993, the Ambros group identified first of miRNA in the nematode C.elegans [26]. MiRNAs are sometimes produced as a single lengthy transcript termed a cluster that may have comparable seed regions and is referred to as a family [27]. MiRNAs are single-strand RNAs with 21–25 nucleotides that are made by hairpin-shaped predecessors. MiRNA genes are produced to a primary miRNA(pri-miRNA) in mammals. Drosha, a class 2 RNase III enzyme, converts pri-miRNA to a precursor miRNA (pre-miRNA) in that nucleus. Exportin-5 (EXP-5) then facilitates the transfer of pre-miRNAs to the cytoplasm. They are then produced in the cytoplasm by Dicer, an RNase III-like protein, to become mature miRNAs, which are then loaded onto the Argonaute (AGO) protein to form the effector RNA-induced silencing complex (RISC) [28] (Fig. 1). MiRNAs have been implicated in various cancer-related biological processes, including tumor proliferation, metastasis, angiogenesis, and drug resistance [29]. Others have been suggested as possible biomarkers for a variety of cancers, including BCa [30, 31]. The aim of this article is to discuss the various functions of miRNAs in the progression of BCa through angiogenesis, and study several novel delivery carriers for miRNA-based therapeutics.

## Cellular and molecular pathways of angiogenesis of breast cancer

### History of research on angiogenesis

Goldman was the first to study tumor vascularization in details, describing the vasoproliferative reaction of the organ where a tumor grows as follows: Chaotic development disrupts the normal blood arteries of the organs in which the tumor is forming; there is dilatation and spiraling of the afflicted arteries, significant capillary branching, and new vessel development, especially at the advancing boundary [32–34]. The word "angiogenesis" was coined by a British physician named John Hunter in 1787 [35]. Ide et al. proposed in 1939 that tumors produce particular substances that stimulate blood vessel formation [36]. Growing tumors might trigger new capillary development from the host, as Chalkley and Algire observed in 1945 [37]. According to Shubik and Greenblatt in 1968, new blood vessels are generated in any case by the release of a diffusible component that may travel through the pores [38]. Pietro M. Gullino and colleagues discovered in the 1970s that the normal adult mouse breast gland has little, if any, angiogenic capability, but breast carcinomas regularly gain it [39]. The year 1971 was a watershed moment in the history of neoplastic angiogenesis research, when M. J. Folkman presented his theory that the growth of a neoplastic tumor is dependent on the creation of blood vessels [40, 41]. Michael Klagsbrun and Yuen Shing identified a chemical that is mitogenic for endothelial cells from a tumor in 1984 using the chromatography technique [42]. In terms of molecular structure, this molecule was found to be similar to fibroblast growth factor (FGF). D. Gospodarowicz had previously identified it in bovine pituitary gland cells, and it demonstrated mitogenic effects for fibroblasts, extending their lifetime [43]. Ferrara and Rosalind Rosenthal identified a protein separate from FGF with significant capabilities that promote the growth of vascular endothelial cells in 1989, separately from each other. Ferrara termed it VEGF (vascular endothelial growth factor), and it was quickly given an essential function in neoplastic angiogenesis, such as brain tumors [44, 45].

### General angiogenesis

Vascularization, the process of growing blood vessels into a tissue to enhance nutrient supply, results from chemical indicators from tumor cells in a quick increase process and controls tumor increase and progression. Quiescent vasculature, endothelial cell activation, sprout forming, vascular lumen formation, and new vessel stabilization are all stages in the vessel formation process [46–48]. Angiogenic factors, Platelet-derived growth factor-BB (PDGF-BB), presence of VEGF and Notch signaling pathway, activation of particular steroid hormone receptors, estrogen, progesterone, the family of tyrosine kinases (EGFR, HER1, HER2, HER3, HER4), breast cancer development, endothelial cell migration, proliferation, differentiation, and hematogeneous spread (via the shaped vasculature) are all triggered by angiogenic mediators (Tissue inhibitors of metalloproteinases-1(TIMP-1), Angiopoietin-2(Ang-2))[49–51].

The activation of angiogenesis is triggered by certain compounds known as angiogenic activators, which can stimulate cell proliferation in vitro [52]. Within the tumor microenvironment, signaling occurs between tumor cells and a variety of other cell types. The development of pro-angiogenic growth factors by tumor cells, which influence the existing vessels, is required to induce this mechanism [53–55].

### Stages of angiogenesis

(1) Hypoxia activates hypoxia-inducible factor-1 (HIF-1), which leads to the production of pro-angiogenic elements. The most important of which being VEGF (vascular endothelial growth factor). VEGF plays a role inactivator of endothelial cell proliferation and migration. (2) Hypoxia also causes basement membrane breakdown and pericyte separation by up-regulating protease expression. (3) Angiogenic factor gradients drive the migration of specific endothelial cells known as “tip cells”. (4) Endothelial cells develop into proliferative stalk cells, which form the new vessel’s main body. (5) VEGF increases DLL4 (Delta-like ligand 4) secretion, which interacts with Notch-1 receptors and suppresses proliferation by downregulating vascular endothelial growth factor receptor (VEGFR). (6) Pericyte attachment is stimulated by Platelet-derived growth factor (PDGF), which also inhibits proliferation and sensitivity to VEGF. Tumor development is aided by an increase in blood flow [56, 57] (Fig. 1).

A dynamic signal equilibrium between anti- and pro-angiogenic elements is actively maintained in the microenvironment during these closely controlled cycles, in order to build and strengthen newly developed blood vessels [58]. Tumors trigger the angiogenic transition, according to scientists, by disrupting the equilibrium between angiogenesis inducers and inhibitors. This transition can be made by altering gene trans. On the other hand, cription, as seen in many tumors where VEGF and/or FGF levels are higher than in healthy tissue. The levels of endogenous inhibitors are decreased in other cancers [59, 60]. Angiogenesis in a tumor and the subsequent formation of endothelial tubes is a multi-stage procedure that is controlled by hypoxia at each point. This mechanism is heavily reliant on endothelial cells (ECs) expressing the HIF-1 heterodimeric transcription factor [61, 62]. In the presence of hypoxia, the protein HIF-1 has been stabilized and forms a heterodimer with HIF-1β, and this duo stimulates the transcription of multiple target genes in human cancer cells to respond to the hypoxic setting [63, 64].

### Angiogenesis in breast cancer

As previously mentioned, in cancer metastasis pathological angiogenesis plays a critical role [65–67]. Angiogenesis is the coordinated modulation of vascular growth factors, including basic fibroblast growth factor (bFGF), transforming growth factor (TGF-1), platelet-derived endothelial cells growth factor, placenta growth factor, and others. Clinical trials have shown that angiogenesis is essential for BCa progression and metastasis [68].

Although a healthy human mammary gland has been found to express minimal levels of protein which the most significant of these growth factors in BCa has been identified as VEGF and its different isoforms [69]. The most studied growth factors are VEGF and interleukin-8 (IL-8). BCa cell lines that express a lot of VEGFS also express a lot of IL-8. They appear to play a critical role in the promotion of angiogenesis in BCa [70, 71]. Invasive BCa has been shown to have an elevated level of VEGF receptor-3, which has also been found to be up-regulated in the endothelium of angiogenic blood vessels [52]. The relationship between VEGF-A and VEGFR-1 or 2 is vital in the growth, progression, and metastasis of BCa [72].

Increased ranges of angiogenic boom elements in BCa cells had been related to the aggressiveness and hazard of invasive BCa [73]. This has also been connected to the inactivation of the p53 gene [74]. In addition, the presence of micro-vasculature in surgical samples of invasive BCa might indicate metastasis or recurrence. In tumors, angiogenesis requires the interaction of one or more of these growth agents. According to studies: VEGF, IL-8, bFGF/FGF-2, and Matrix metalloproteinases (MMPs), enhance the formation and metastasis of tumor[75]. Macrophages, airway smooth muscle cells, tumor cells, and other cell types all express IL-8, a member of the chemokine family, have been shown to stimulate VEGF activity in endothelial cells (ECs) through binding to its receptor [76, 77].

IL-8 also significantly affects angiogenesis by promoting the proliferation and longevity of ECs and up-regulating MMPs in some endothelial cell lines and in vitro stimulation of capillary tube development [78]. These are all important characteristics of breast cancer development and metastasis. Breast tumors with high IL-8 levels have been shown to be more aggressive and invasive, rendering IL-8 levels a promising option for anti-angiogenic therapies, as well as a possible prognostic biomarker for a number of tumors, including BCa [79]. Furthermore, data from human and mouse models of cytomegalovirus infection in cancers including glioblastoma and breast cancer suggest that the virus can alter epithelial cells, enhance tumor angiogenesis and proliferation, and suppress the host anti-cytomegalovirus immune response [80]. Fibroblast growth factors (bFGF/FGF-2) are a group of potent angiogenic stimulators that have been related to an increased risk of BCa [81]. Substances can regulate angiogenesis, tumor proliferation, and metastasis in the extracellular environment via modulating the interactions between FGF-2 and its receptor [82]. Also, MMPs are part of a broader class of proteases involved in angiogenesis due to their ability to destroy extracellular matrix proteins and thereby remodel the extracellular matrix [83].

## The role of microRNAs in angiogenesis

### Biogenesis of miRNA

**Fig. 2:**
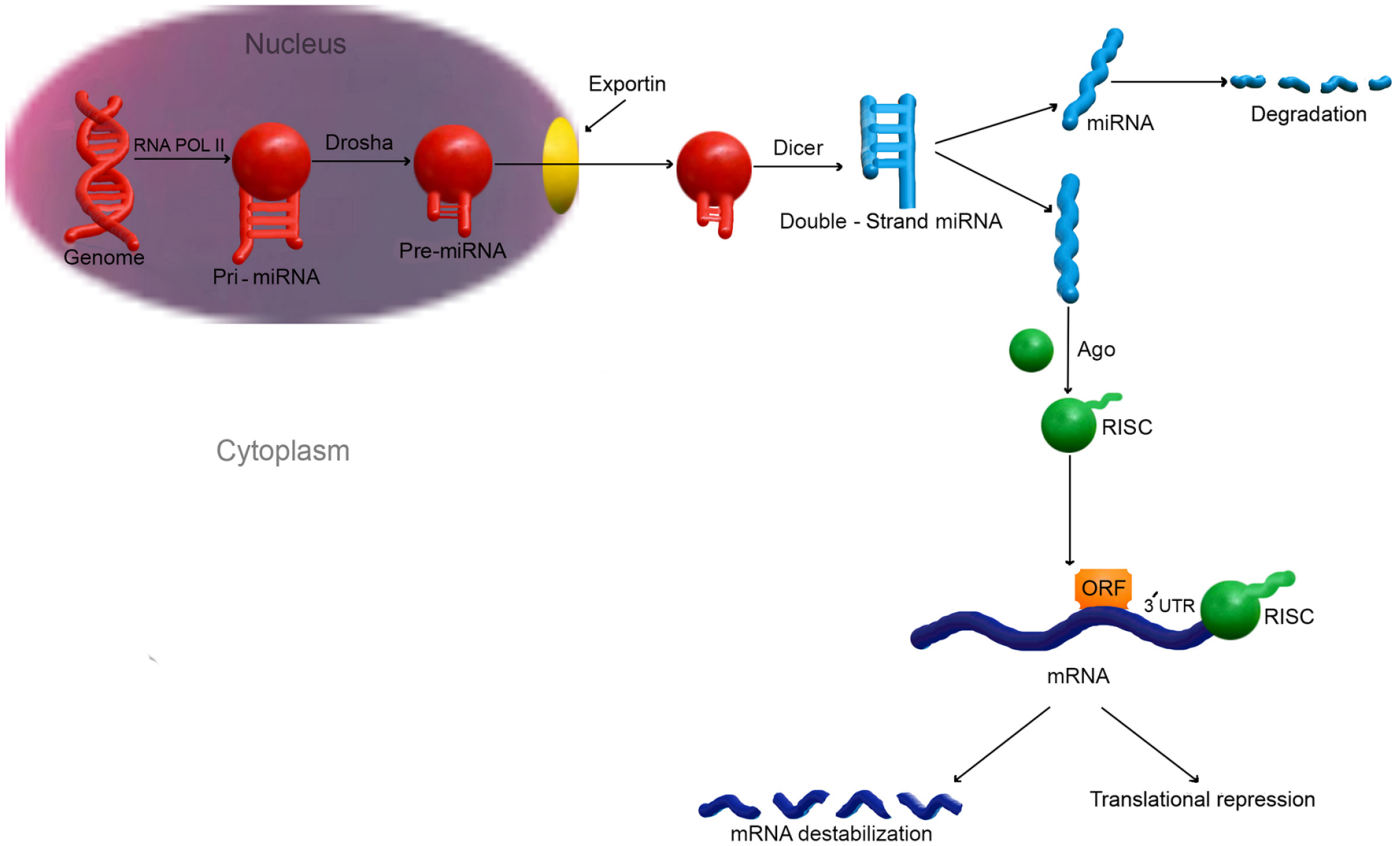
MicroRNA biogenesis RNA polymerase II transcribes intergenic microRNA genes into locally hairpin formations called the primary microRNAs (pri-miRNAs), that are then properly processed to precursor microRNA (pre-microRNA) via Drosha and Pasha in the nucleus. Owing to Dicer activity in conjunction with additional cofactors, a 22 nt duplex is produced after exporting in the cytoplasm. MicroRNAs that have been mature recognize seed sequences in the 3′ UTRs of target mRNAs and regulate gene expression by interrupting or inhibiting target mRNA translation

In 1993, Ambros et al. identified first miRNA in the nematode C. elegans [26]. According to an early study, the miRNAs are predominantly found in intergenic regions, and small amount of them are found in intronic regions [84]. RNA polymerase II (Pol II) is majorly responsible for miRNA gene transcription [85, 86]. However, RNA polymerase III (Pol III) can transcribe a select number of genes related to Alu repeats. The primary miRNA (pri-miRNA), which is normally several kilobases long and contains local stem-loop structures, is the result of Pol II or Pol III-mediated production [87]. The stem of the hairpin of the pri-miRNA generated by Pol II is broken, releasing a 60–70 nucleotides (nt) hairpin structure is called the precursor miRNA (pre-miRNA) [86, 88]. Drosha, that in humans needs the DiGeorge syndrome critical region in gene 8 (DGCR8) and Pasha in C. elegans or D. melanogaster as a cofactor, performs this function [89]. Drosha produces a huge complex known as the microprocessor complex when combined with either DGCR8 or Pasha [90]. Drosha is guided by DGCR8 to slice pri-miRNA by interacting with the single-stranded RNA (ssRNA) segment. Drosha converts the pri-miRNA to the pre-miRNA with a 5′-phosphate end and a roughly 2 nt 3′ overhang by cleaving RNA duplexes around 11 base pairs distant from the ssRNA-stem loop connection [89, 91]. Pre-miRNAs are carried into the cytoplasm, where they are processed into mature miRNAs. Nuclear pore complexes, which become giant proteinaceous channels located in the nuclear membrane, are where the pre-miRNA is transported [92]. The RanGTP-dependent nuclear transportation receptor EXP5 is responsible for the transport of pre-miRNA [93, 94]. EXP5 releases the pre-miRNA into the cytoplasm, where it is cleaved by Dicer, a cytoplasmic RNase III endonuclease [95, 96]. Due to the present paradigm, the miRNA duplex is integrated into an AGO family protein complex following Dicer cleavage generates about 22 nt miRNA duplex. This results in the formation of an effector complex. The miRNA is mostly destroyed on one strand (passenger strand or miRNA), while the other strand remains linked to AGO as mature miRNA (guide strand or miRNA). However, in a few circumstances, miRNA is transported into RISC and hence continues to operate [97]. Recent data indicate that the thermodynamic stabilization of the duplex's two ends may influence which strand is chosen [98] (Fig. 2).

### MiRNAs in angiogenesis

MiRNAs have been implicated in many different ways such as cancer-related biological processes, in particular, They influence the genes involved in the angiogenic switch at the post-transcriptional level [99, 100]. MiRNA binds to the 3UTRs of mRNAs; due to this, translation is degraded or blocked in a wide range of biological activities [101, 102]. MiRNAs control the manufacturing of pro- or anti-angiogenic molecules in tumor cells, as a result, the proliferation and migration of ECs are regulated in a paracrine way [103]. Over four hundred miRNAs have been discovered in the genome of the human, which EC function and angiogenesis have been proven to be targeted in 10% of the cases. MiRNAs have a high level of expression in ECs, which is linked to angiogenesis control [104, 105]. Generally there are two types of miRNAs: miRNAs that target angiogenesis-related genes with (1) pro-angiogenic effect (2) anti-angiogenic effect [106]. Moreover, the same miRNA may sometimes have dual effects by targeting different genes. miR-126 is example of this type of miRNAs, which has pro/anti-angiogenic effects in different cancer [107] (Table 1).

**Table 1:** An overview of miRNAs linked to angiogenesis

miRNA	Angiogenesis’ major function and role	References
miR-34a	Anti-angiogenesis in endothelial cells and tumors	[298, 303]
miRNA-29	Anti-angiogenesis in endothelial cells and tumors	[114, 116, 304, 305]
miRNA-124	Anti-angiogenesis in endothelial cells and tumors	[306, 307]
miR-19	Anti-angiogenesis in endothelial cells	[308]
miR-92a	Anti-angiogenesis in endothelial cells	[157]
miR-206	Anti-angiogenesis in tumors	[309]
miR-483	Anti-angiogenesis in endothelial cells	[310, 311]
miR-16 family miR-15b, miR-16	Anti-angiogenesis in tumors and endothelial cells	[168, 312]
miR-26	Anti-angiogenesis in tumors and endothelial cells	[138, 313]
miR-17–92 miR-18a	Pro-angiogenesis in tumors and endothelial cells Pro-angiogenesis in tumors and anti-angiogenesis in endothelial cells	[71, 156, 308, 314]
miR-210 miR-21	Pro-angiogenesis in tumors and endothelial cells Pro-angiogenesis in tumors and endothelial cells	[121, 315, 316]
miR-296	Pro-angiogenesis in tumors and endothelial cells	[126]
miR-130a	Pro-angiogenesis in tumors and endothelial cells	[128, 129]
let-7	Pro-angiogenesis in endothelial cells	[132, 317]
miR-155	Pro-angiogenesis in tumors and endothelial cells	[318, 319]
miR-150	Anti-angiogenesis in tumor s pro-angiogenesis in endothelial cells	[160, 161]
miR-126	Anti-angiogenesis in tumor pro-angiogenesis in endothelial cells	[142, 145, 320]
miR-221/222	Anti-angiogenesis in endothelial cell pro-angiogenesis in VSMCs and tumor s	[148, 321–323]
miR-424	Pro-angiogenesis in endothelial cells during hypoxia and ischemia Anti-angiogenesis in endothelial cells without hypoxic and in tumors	[173, 324, 325]
miR-93	Dual-action in angiogenesis in both endothelial cells and tumors	[164, 167]
miR-27b	Dual function in angiogenesis in both endothelial cells and tumors	[177, 180]
miR-9,miR-135a, miR-181a, miR-181b, miR-199b,miR-204	Managing angiogenesis by targeting SIRT1	[181]
MiR-200b,miR-361-5p, miR-874,miR-125- 5p, miR-146	Managing angiogenesis by targeting VEGF	[182]

### MiRNAs that target angiogenesis genes

#### miRNA-34a

In coronary artery disease, miRNA-34a expression is considerably higher. However, it is diminished in prostate and BCa cells that are CD44- or CD133-positive [108, 109]. Angiogenesis can be hampered by miRNA-34a. Furthermore, excessive miRNA-34a expression inhibits tumor angiogenesis, significantly. The modulation of miRNA-34a activity offers a new treatment method for prostate, HNSCC, and breast cancer therapy [110]. In nasopharyngeal cancer, an enhancer of Zeste Homolog 2 enhances angiogenesis by inhibiting the miR-1/Endothelin-1 axis [111]. It was also discovered that miR-648 targets Endothelin-1 in gastric cancer cells [112]. In addition, miR-34a downregulation in ankylosing spondylitis was linked to survivin overexpression, which is implicated in angiogenesis [113].

#### miRNA-29

miRNA-29 is made up of three different miRNAs: miRNA-29a, b, and c [114]. In diabetic MMEVC, inhibiting miRNA-29 can improve angiogenesis by encouraging cell proliferation through elevating IGF1. It's possible that miRNA-29 inhibits angiogenesis in ECs. Endometrial carcinoma, gastric cancer, hepatocellular carcinoma (HCC), and BCa all have low levels of miR-29a/b/c [115]. By targeting VEGFA through the PI3K/AKT and MAPK/ERK signaling pathways, miRNA-29b can inhibit angiogenesis in endometrial cancer. In therapeutic applications, miRNA-29a/b/c recovery treatment has much potential. However, further research is needed to completely comprehend the roles and processes of miRNA-29 in many disorders [116–118].

#### miRNA-124

The mature versions of miRNA-124 are miRNA-124-3p and miRNA-124-5p. In BCa cells, miRNA-124 suppresses angiogenesis and proliferation via targeting ETS-1 and AKT2 [119]. Furthermore, increasing miR-124-5p levels can suppress angiogenesis. MiR-124 has been shown in studies to be used as a therapeutic target for malignancies [120].

### Angiogenesis-inducing microRNAs

#### miRNA-21 and miRNA-210

MiRNA-21 enhances the production of VEGF, which increases tumor angiogenesis [121]. MiRNA-210 enhances the expression of VEGF, Notch, and VEGFR-2 in human umbilical vein endothelial cells (HUVECs), promoting angiogenesis and vasculature development in post-ischemic brain tissue [122]. MiRNA-210 suppression promotes apoptosis and autophagy in tumor cells while suppressing angiogenesis. MiR-210 is the most consistently and significantly induced miRNA during hypoxia, affecting cell survival, migration, and differentiation. it can facilitate the capillary sprouting formation and decrease apoptosis by inhibiting Ephrin-A3 under hypoxic conditions [123]. Also, it has been shown that overexpression of miR-210 is induced by hypoxia in a HIF-1α– and von Hippel-Lindau (VHL)-dependent manner and its expression levels are an independent prognostic factor in breast cancer samples [124]. As a result, miRNA-210 might be a viable target for supplemental therapeutic therapy of malignancies and ischemic illnesses, as well as a possible prognostic and diagnostic biomarker for determining tumor aggressiveness [125].

#### miRNA-296

Angiogenesis is caused through miRNA-296 expression in vascular sickness and cancer [126, 127]. In ECs, growth factor-mediated or glioma miRNA-296 will increase ranges of receptors for pro-angiogenic growth factors, consistent with research. Furthermore, manipulating miRNA-296 levels might have therapeutic implications in tumor development and angiogenic diseases, where angiogenesis is a key factor [126].

#### miRNA-130a

MiRNA-130a inhibits EC proliferation and metastasis, as well as tube formation, by inhibiting the anti-angiogenic homeobox proteins GAX and HoxA5 [128]. By targeting tissue factor pathway inhibitor 2 (TFPI2), a miRNA-130a inhibitor inhibits haemangioma development and angiogenesis. A miRNA-130a inhibitor might be a helpful cancer therapy method. Nonetheless, it is concerning that using miRNA-130a which mimics to treat ischaemic diseases can raise the risk of developing tumors and using miRNA-130a inhibitors to treat cancers can end result in ischemia diseases [129].

#### miRNA-155

Many forms of human malignancies and vascular disorders have been demonstrated to up-regulate miRNA-155, according to mounting data. According to recent reviews, miRNA-155 promotes tumor angiogenesis by triggering the down-regulation of von Hippel- Lindau (VHL) and knocking down miRNA-155 decreases HUVEC proliferation, migration, and network development [130, 131]. For angiogenesis-related diseases, miRNA-155 might be a valuable prognostic indicator as well as a therapeutic target.

#### miRNA-let-7

Let-7f mimics boost the number, proliferation, migration, and network development of pro-angiogenic cells (PACs). In the clinical therapy of Deep Vein Thrombosis (DVT), miR-let-7e-5p might be a potential therapeutic target. Finally, employing miRNA mimics as a therapeutic method to promote angiogenesis in ischemic disorders might be a game-changer [132, 133].

### Angiogenesis-inhibiting miRNAs

#### miRNA-483

Hypoxia reduces the expression of MiRNA-483-5p. In a rat model of thrombosis, Kong et al. discovered that miRNA-483-3p may suppress angiogenesis and is up-regulated in EPCs of DVT patients, limiting EPC migration and tube formation and accelerating apoptosis in vitro by targeting SRF, thereby lowering EPC homing and thrombus organization and recanalization. Inhibition of miR-483 improves HUVEC angiogenesis, according to these findings. As a result, miR-483 may be used to treat ischemic diseases [132].

#### miRNA-206

According to the findings, MiRNA-206 inhibits VEGF-mediated angiogenesis in TNBC and Non-small-cell lung carcinoma (NSCLS) and this is greatly diminished under hypoxic conditions [134]. In addition, in NSCLC, miRNS-206 inhibits proliferation, tube formation, growth, and angiogenesis. MiRNA-206 might be a powerful target for inhibiting the development and angiogenesis of many malignancies by regulating many targets [135].

#### miRNA-26

MiRNA-26 is made up of two miRNAs: miRNA-26a and miRNA-26b. Pro-angiogenic stimuli like VEGF and TNF can cause MiRNA-26a to be downregulated [136]. MiRNA-26a inhibits angiogenesis by inhibiting proliferating, mobility, and tube formation in HUVECs via mechanically repressing VEGF signaling by specifically targeting NgBR [137]. Furthermore, gastric cancer and HCC have lower levels of miRNA-26a/b, which can block angiogenesis and diminish gastric cancer proliferation and migration [138, 139]. In HCC cell lines and tissues, miRNA-26b-5p is clearly downregulated, inhibiting angiogenesis and repressing apoptosis and tube formation. These findings suggest that miRNA-26a/b can inhibit angiogenesis in ECs and tumors via a variety of mechanisms, making it a potential new biomarker and target for the management and therapy of angiogenesis-related illnesses [140].

### Angiogenesis miRNAs with a double function

#### miRNA-126

miRNA-126 is mostly found in vascular ECs, which is strongly linked to angiogenesis in both normal growth and damage repair [141, 142]. There are two type of the pre-miRNA-126: (1) miRNA-126-3p (2) miRNA-126-5p. MiRNA-126-3p enhances EC angiogenesis By inhibiting negative regulation of the pathways [143]. MiR-126-5p promotes angiogenesis and protects atherosclerosis by suppressing the Notch1 inhibitor Delta Like Non-Canonical Notch Ligand 1 (DLK1) [143, 144]. Furthermore, miRNA-126-3p reduces LRP6 and PIK3R2,59, which decreases angiogenesis in hepatocellular carcinoma (HCC). As a result, miRNA-126 seems to be a tumor angiogenesis inhibitor. Because the signaling mechanisms involving miRNA-126 in angiogenesis regulation are currently poorly understood, the mechanisms of miRNA-126 must be investigated further to establish a novel therapeutic target for treating and preventing angiogenesis-related illnesses [145].

#### miRNA-221 and miRNA-222

MiRNA-221 and miRNA-222 are highly similar [146, 147]. Many studies have shown that miRNA-221 and miRNA-222 promote and/or decrease tumor angiogenesis in gastric cancer [148], HCC [149], lung cancer [150], BCa [151], human glioblastoma [152], human epithelial malignancies [153], and other malignancies. Other studies of miRNA-222 and miRNA-221 are expected to provide further information on the complex miRNA. MiRNA-based therapeutic breakthroughs may become a viable therapy option for incurable malignancies if they are further developed [154, 155].

#### miRNA-17–92 cluster

MiRNA-17-3p, miRNA-17-5p, miRNA-18a, miRNA-19a, miRNA-19b, miRNA-20a, and miRNA-92a are among the miRNAs studies [156, 157]. Angiogenesis is enhanced by miRNA-19 and miRNA-18. MiRNA-17 inhibits angiogenesis in ECs via inhibiting numerous targets. Intriguingly, inhibiting miRNA-20a and miRNA-17 promotes angiogenesis in ECs while having no effect on tumor angiogenesis. Meanwhile, new information about the miRNA-17-3p, miRNA-17-5p, miRNA-18a, miRNA-19a, miRNA-19b, miRNA-20a, and miRNA-92a will help researchers to gain knowledge of the complex control of angiogenesis and will aid in the evolution of a more practical, targeted miRNA-based treatment strategy in the therapy [158].

#### miRNA-150

In atherosclerotic settings, down-regulation of miRNA-150 impairs angiogenesis and blood flow after ischemic tissue restoration, according to a recent study [159]. MiRNA-150 overexpression in ECs enhances cell motility and angiogenesis. These findings showed that miRNA-150's impact varies depending on the cell type and pathological circumstances [160–162].

#### miRNA-93

In numerous molecular mechanisms, miRNA-93 stimulates and/or suppresses angiogenesis. Several studies showed that miRNA-93 promotes angiogenesis and improves EC growth, motility, dissemination, and tube formation. By inhibiting homology 2 (LATS2), increased miRNA-93 expression increases breast cancer angiogenesis [163, 164]. However, miRNA-93 appears to have a function in the inhibition of angiogenesis in some pathological conditions [165]. Angiogenesis and colorectal cancer development are inhibited by miRNA-93, according to studies [166]. MiRNA-93 inhibits angiogenesis induced by IL-8 and VEGF in neuroblastoma cells. Furthermore, in human lymphatic endothelial cells (HLECs), miRNA-93 inhibits angiogenesis. Through several molecular pathways, miRNA-93 has double effects on angiogenesis in many cells and tissues. The intricate system of miRNA-93 that affects angiogenesis in many tumors and vascular disorders will require further exploration in the future [167].

#### The miRNA-16 family

miRNA-497, miRNA-424, miRNA-195, miRNA-16, miRNA-15a and miRNA-b are all members of the miRNA-16 family. According to one study, hypoxia-induced loss of miRNA-16 and miRNA-15b results in an increase in VEGF. Over-expression of miRNA-16 and miRNA-15 may also be a promising anti-tumor therapy that inhibits VEGF-mediated angiogenesis and also reduces tumor cell proliferation [168]. MiRNA-15b inhibits brain tumor angiogenesis, according to research findings. As a result, miRNA-15b might be a potential anti-angiogenesis research focus [169, 170]. Through reducing the levels of cell division cycle 42 (Cdc42), interaction of FGF1 with the VEGF, and Cdc42 3UTR, miRNA-195 decreases vascular smooth muscle cells (VSMC) proliferation and migration, repressing angiogenesis [171]. Furthermore, miRNA-195 downregulation enhances VEGF in the tumor microenvironment, which stimulates VEGFR2 signaling in ECs and improves angiogenesis. As a result, miRNA-195 plays a vital role in the biological functions of angiogenesis, and it is a promising therapeutic target for inhibiting angiogenesis [172]. Under hypoxia, miRNA-424 is significantly elevated. During hypoxia, miRNA-424 and miRNA-322 dramatically increase EC proliferation and metastasis, and they additionally induce angiogenesis in vitro. Overexpression of miRNA-424, Nevertheless, inhibits angiogenesis [173]. Furthermore, miRNA-424 stops ECs from proliferating, migrating, and forming tubes [174]. In addition, miRNA-424 inhibits angiogenesis by lowering the amounts of VEGFA protein in endometriotic cells and endometrial. In conclusion, miRNA-424 has two impacts on angiogenesis, although in the majority of situations, it stimulates angiogenesis [175].

#### miRNA-27b

In recent years, a growing large number of studies have revealed that the roles of miRNA-27b in various malignancies and vascular illnesses are fundamentally distinct [176, 177]. MiRNA-27b enhances angiogenesis in type 2 diabetic mice with deficient bone marrow-derived angiogenic cells (BMAC) in vivo and by directly suppressing the manufacturing of p66 (shc), Semaphorin6A (Sema6A), and thrombospondin-1 (TSP-1) in vitro [177]. Also, miRNA-27b expression levels correlate with the metastatic state of TNBC [178]. Furthermore, miRNA-27b promotes the proliferation and metastasis of ECs. As a result, angiogenesis is aided [176]. In mice with MI, miRNA-27b also improves angiogenesis and ejection percentage [179]. However, in certain circumstances, miRNA-27b suppresses angiogenesis. Over-expression of miRNA-27b inhibits the VEGFC/VEGFR2 signaling pathway, which lowers HUVEC growth, motility, and tube formation, as well as angiogenesis in colon cancer & gastric cancer [176]. Studying the exact processes of miRNAs is thus a huge problem. A closer look into the control mechanisms of angiogenesis by miRNA-27b in angiogenesis-related disorders might lead to new miRNA-based therapeutics [180].

### Angiogenesis's other miRNAs

Angiogenesis may be controlled by miRNA-181a, miRNA-135a, miRNA-199b, miRNA-181b, miRNA-204 and, miRNA-9, according to studies [181]. By modulating VEGF miRNA-125-5p, miRNA-200b, miRNA-361-5p, miRNA-146, and miRNA-874 have a role in angiogenesis [182]. Furthermore, angiogenesis caused by Ginsenoside-Rg1, a predominant protopanaxatriol-type ginsenoside in *P. ginseng*, is mediated through the miR-214/eNOS mechanism [183].

## microRNAs in the therapy of breast cancer angiogenesis

It is illustrated that MiRNAs have impressive potential as the quality asset for the treatment of BCa. ECs are one of the key components of the tunica intima, playing imperative parts in tumor angiogenesis. It has appeared that numerous miRNAs such as miRNA-330, miRNA-216a, and miRNA-608, which can tie to both the CD44 and CDC42 (CD44 downstream target mRNA) 3'-UTRs, can balance EC exercises [184]. MiRNA-126, which is especially associated to vascular ECs, has an imperative part in smothering BCa angiogenesis through focusing on PITPNC1, IGFBP2, and MERTK [185] and controlling the VEGF/PI3K/AKT signaling cascade [186]. Further, miRNA-9 contributes to angiogenesis via the upregulation of VEGF expression in BCa [187]. One of the novel strategies to cancer therapy is utilizing of chimeric antigen receptor (CAR) T cell therapy, which numerous efforts have been performed in this field [188–190]. Scientists have considered using anti-angiogenic CAR-T cells to treat cancer. They created CARs with a strong affinity for VEGFR2, which is expressed on both cancer cells and endothelial cells. As a result, anti-VEGFR2 CAR-T cells function as a dual-purpose targeted treatment that targets both tumor cells and ECs [191]. Another document has illustrated that VEGF expression in breast cancer cells intercedes through HIF-1, flag transducer and activator of transcription3 (STAT3) in a miRNA-20b-structured way [192]. The overexpression of miRNA-10b and miRNA-196b has been diagnosed in the vasculature of high-grade BCa. The most important thing is to be aware of the incitement of VEGF [194]. The results indicate that miR27a exhibits oncogenic activity through regulation of ZBTB10, which in turn leads to the overexpression of Sp proteins and Sp-dependent genes, which are important for cell survival and angiogenesis [194]. The overexpression of miRNA-19 downregulates tissue factor (TF) expression, which is a vital factor inside the course of tumor angiogenesis, presenting the ability of miRNA-19 for BCa angiogenesis. Altogether, the inclusion of those miRNAs inside the pathogenesis and motion of BCa has been elucidated, that some of them performing as silencers and others as tumor promoters. These cases are pivotal to assess their application as medical biomarkers and their beneficial potential [194].

## Development of the carriers for efficient delivery of miRNA-based therapy

We mentioned in the previous section that numerous studies on miRNA-based therapeutics have been done as a result of the compelling evidence for the function of miRNAs in the angiogenesis but nowadays, efficient miRNA delivery is required for using miRNAs to treat cancer effectively [195]. For this purpose, delivering anti-miRNA oligonucleotides (anti-miRs) against oncogenic miRNAs or tumor-suppressor miRNA mimics are the two major approaches that can deliver directly to target cells with different strategies [196, 197]. The miRNA delivery is more preferable than antibody delivery due to the ability of miRNAs to induce or inhibit several downstream genes. We discuss about these delivery strategies in the following.

### Viral-based carriers

Viral vectors are especially effective for delivering miRNA genes to specific target cells and are among the most vital instruments used in the treatment of illnesses. Numerous viral vectors have been developed and improved to this point, and each one has unique properties that make it better suited for a particular use than others [198, 199]. The first one is adenoviruses (ADVs) which are non-enveloped viruses from the Adenoviridae family with linear double-stranded DNA (dsDNA) [200]. The simplicity of genetic manipulation is one of the important advantages of this vector. Moreover, they have the ability to trigger immunological reactions that might hinder effective gene transfer and the recycling of ADV vectors and cannot replicate and integrate with the host genome [201–203]. Several studies have shown that the large load capacity of these vectors is one of their main features, and they have drawn specific attention for concurrent transport of protein and miRNA [204, 205]. In regard to breast cancer Kim and colleagues reported that Ad-miR-145, a miR-145 developed as an ADV, was delivered to breast cancer cells and reduced cell proliferation and movement in both in vitro and in vivo systems [206]. The next one is Adeno-associated viruses (AAVs) which are a group of non-enveloped viruses with linear single-stranded DNA (ssDNA) [207]. The main advantages of AAVs as a vector are their lack of pathogenicity and failure to induce cancer in individuals (in the wild-type of the virus) [208]. But so far it has not been used in the delivery of miRNAs in breast cancer. Retroviruses and Lentiviruses (a subgroup of retroviruses) are a family of enveloped viruses containing single-stranded RNA (ssRNA) [209–211]. The reverse transcriptase enzyme is used by these viruses to produce the DNA form of their genome. The integrase enzyme inserts the produced DNA into the genome of the host cell. This ability of retroviruses is extremely beneficial for the stable expression of target genes [196, 198, 209]. According to studies, intravenously administered lentivirus with miR-494 antagonists dramatically decreases the number of myeloid-derived suppressor cells (MDSCs) that infiltrate tumors in a murine breast cancer model and inhibits tumor development and metastasis [212]. Nowadays, based on recombinant viral capsids, virus-like particles (VLPs) are a recently developed technique that can be utilized to deliver therapeutic miRNAs in the treatment of systemic lupus erythematosus (SLE) [213].

### Non-viral based carriers

Despite being extremely effective, viral-based miRNA delivery methods have significant immunogenicity, toxicity, and size restrictions. There are now non-viral methods of miRNA distribution such as lipid-based, polymer-based, liposome-based, and inorganic vectors that are less harmful and biocompatible that can address these problems. Additionally, compared to viral vectors, non-viral methods are more affordable to produce [214, 215].

### Lipid-based vectors

The most prevalent non-viral delivery techniques are lipid-based carriers [216]. Lipids have played a vital role in the development of innovative drug delivery methods ever since the first liposomes were developed, so that, liposomal drug delivery is an important area of previous studies [217, 218] and today liposomes are used to encapsulate nucleic acids and transfer them to cells. Lipoplexes are complexes formed when negatively charged miRs attach to positively charged lipids (cationic lipids), which is how positively charged lipids are often employed for miR distribution [219]. In Li et al. research, it has been demonstrated that miR-34a may significantly decrease breast cancer cell proliferation, migration, and invasion when miR-34a target genes are downregulated as a consequence of delivery of a plasmid expressing miR-34a (T-VISA-miR-34a) through cationic liposomes to cultured breast cancer cells. Moreover, intravenous administration of this complex nanoparticles dramatically reduced tumor development, prolonged survival, and did not result in systemic toxicity in an orthotopic animal model of breast cancer [220–222]. Moreover, to reduce toxicity due to untargeted miRNA delivery therapy, the usage of antibodies can be helpful. The ability of the nanoparticles to target tumor sites further improves the therapeutic value of RNA therapeutics [223–226]. The capacity to deliver miRNA with simultaneously targeting different oncogenic pathways is definitely an important advantage of the usage of antibody-based delivery approaches. In this regard, Chen et al. developed a LPH (liposome-polycation-hyaluronic acid) nanoparticle formulation modified with tumor-targeting scFv for delivery of miR-34a into experimental lung metastasis of murine model of melanoma. They revealed that miR-34a delivered by GC4-targeted nanoparticles significantly reduced tumor load in the lung and downregulated expression of survivin in the metastatic tumor [227]. Survivin, an anti-apoptotic factor that has been identified as a therapeutic target in a variety of disorders, plays a crucial part in mechanisms that promote tumor development and angiogenesis [228]. HIF1α stability in low oxygen environments and/or through ROS generation enhances survivin and VEGF expression and angiogenesis [229, 230]. In another study, to deliver anti-miR191 to breast cancer cell lines, cationic liposomes based on stearylamine (SA) were developed. As demonstrated in vitro in breast cancer cell lines, treatment with SA liposomes loaded with anti-miR-191 and anti-cancer medicines significantly increased apoptotic cell death and inhibited the migration of cancer cells [231]. According to Bayraktar et al. research, liposomal miR-603 is a tumor suppressor molecule that inhibits the growth and invasion of TNBC both in vitro and in vivo [232]. Recently, Lujan et al. also described the development and manufacturing of nanometer-sized liposomes for the transport of miRNA, with up to 40-fold improvements in miR-203 distribution to MDA-MD-231 breast cancer cells [233].

### Polymer-based vectors

The use of polymeric carriers in medication delivery has a long and promising history. Numerous studies have shown that polymer-based carriers may carry active substances in vivo to the appropriate tissue while protecting them from degradation and improving their water solubility [208, 217, 234, 235]. Two of the most widely used polymeric carriers of nucleic acids are polyethyleneimine (PEI) and poly (lactic-co-glycolic acid) (PLGA) [215]. PEI has been widely used as the foundation for gene delivery since 1995 as a result of its high transfection effectiveness [236–238]. Because nucleic acids are negatively charged, the polymer's positive charge is appropriate for condensing with them to create polyplexes and in general, the PEI is recognized for its remarkable ability to bind to miRs and transfect cells [239, 240]. For example, an anti-miR-155 polyplex has been created using disulfide cross-linked polyethyleneimine (PEI-SS) for the treatment of breast cancer. In this research have been shown that high intracellular glutathione concentration causes the fast release of the miRNA therapies from the polyplex by rupturing the disulfide bonds after endocytosis has allowed it to enter the cells and efficiently inhibited tumor growth in vivo [241]. Earlier studies shown that miR-21 gave BC cells a strong proliferative capacity, a low rate of apoptosis, and a high capacity for invasion and for this reason, Gao and coworkers revealed that delivery of anti-miR-21 with poly(L-lysine)-modified PEI complex (PEI-PLL) to breast cancer cells resulted in an increase in G1 cell cycle arrest and elevated caspase-3 expression, which caused apoptosis [242]. As an alternative, PLGA polymers are also often utilized in miR and drug delivery in general because of their adaptability, biocompatibility, and biodegradability, as well as the creation of stable nanoformulations for sustained drug release [215, 217]. PLGA polymers can also be further modified, for example, with poly ethylene glycol (PEG) to enable prolonged circulation in vivo or with peptides or antibodies to accomplish active targeting to cells. In this regard, in order to treat triple negative breast cancer, Bhargava-Shah et al. created nanoparticles with PLGA-PEG polymer for the delivery of anti-miR-21. They discovered that the treatment made the cells more susceptible to orlistat treatment, which was suggested as a novel repurposing drug for the disease [243]. In a different research, anti-sense miR-21 and miR-10b were successfully delivered by PLGA-PEG in triple-negative breast cancer so that, in vitro cell migration and invasiveness are significantly and incrementally reduced when antisense-miR-21 and antisense-miR 10b combinations are delivered simultaneously [244]. Dendrimers are a different type of polymeric structures that are gaining popularity for use in nucleic acid delivery. Poly amidoamine dendrimer (PAMAM), a dextran-based hydrogel that has been employed in breast cancer therapy for the administration of miR-205 mimics and antagomiR-221, is one application of dendrimers as nonviral vectors for miRNA delivery [245, 246]. Recently, biocompatible, naturally produced polymer-based vectors (Polysaccharide-based) for miRNA delivery, including chitosan, have been used. Chitosan (CS) has a positively charged global structure and is produced by synthesizing D-glucosamine and N-acetyl-D-glucosamine. Additionally, it can facilitate gene uptake and open tight intracellular connections. As a result, it may be a desirable method of gene delivery [247]. A research employing this vector demonstrated that the cationic polysaccharide molecule chitosan, could carry miR-145 to human breast cancer cells (MCF-7 cell line) and silence the production of target mRNAs in vitro and as a potential candidate for novel anti-metastatic therapeutic applications [248]. Additionally, miR-34a was loaded into hyaluronic acid (HA)-CS NPs together with an anti-tumor drug like doxorubicin (DOX) to further examine the tumor-suppressive activity of this miRNA. As a result, it was shown that miR-34a and DOX have stronger anticancer effects (inhibition of tumor growth and migration) on breast cancer cells while B-cell lymphoma 2 (Bcl-2) expression is inhibited, both in vitro and in vivo [249].

### Liposome-based vectors

Liposomes use their charged phospholipid heads groups to interact with the synthesized oligonucleotides contained in their aqueous section. As a result, they form a shielded chamber that is safe from nuclease destruction. Many liposome-mediated miRNA delivery vehicles are now in use, such as the DC-6–140-DOPE-Choleterol liposome [250]. DDAB:cholesterol:TPGS lipoplexes containing pre miR-107 were given to patients with head and neck squamous cell carcinoma and reduced tumor development by 45.2% [251]. Also, the administration of pre-miR-133b lipoplexes comprising DOTMA: cholesterol: TPGS into A549 non-small cell lung cancer was shown to inhibit tumor development [252]. Many researchers have proposed polycationic liposome, hyaluronic acid (LPH) nanoparticles as effective miRNA delivery vehicles [253]. The administration of coupled siRNAs against MDM2, VEGF-A, and cMyc, as well as miRNA34a, into the lung metastasis of the murine melanoma model via systemic delivery of scFv-LPH (single chain antibody fragment modified LPH) nanoparticles reduced tumor progression to about 20% of the control samples [252]. Anti-miR-296 nanoparticles coupled with cyclic RGD, an integrin-binding tripeptide (cRGD-LPH) disrupted blood vessel formation and endothelial cell migration in human umbilical vein endothelial cells via selective uptake through αvβ3 integrin [254]. Although lipid-based miRNA delivery systems have proven to be a reliable platform for miRNA-based therapies, they may provoke hypersensitivity responses due to their toxicity and preferential accumulation in the reticuloendothelial system [255].

### Inorganic-based vectors

In numerous research, inorganic nanoparticles are used for miRNA distribution in addition to lipids and polymers. Gold nanoparticles (AuNPs), carbon nanotubes, silica, nano-diamond and iron oxide-based nanoparticles are a few examples of inorganic vectors for the transport of miRNA [215, 256–260]. They may also be biocompatible, non-immunogenic, nontoxic, and readily made on a big scale [240]. As an example, in vitro delivery of miRNAs to breast and prostate cancer cells was successfully accomplished using AuNPs. In a report by Ekin et al., thiolated RNAs were added to a high-affinity gold nanoparticle-based nanocarrier, and miR-145 was then hybridized to the RNAs bound to the AuNPs. Successful delivery of the AuNP-RNA-miRNA carrier complex resulted in upregulation of miR-145 expression in breast and prostate cancer cells [261, 262]. Further, a nanoparticle made of gold particles and a miR-155 antagonist was developed by Kardani et al. to block the activity of the miR-155 in MCF‐7 breast cancer cell line. Their findings showed a considerable decrease in the levels of this MiRNA and an increase in the mRNA for TP53INP1, which prevented the growth of these tumor cells and encouraged apoptosis [263]. The research by Ramchandani et al. is a recent example of miR-replacement treatment employing AuNP. The authors discovered that alternative multilayers of poly-L-lysine (PLL) and miR-708 mimics were coated on gold nanoparticles. After the PLL layers were broken down by proteases that were overexpressed in tumors, miR-708 mimics were released, which allowed for the restoration of tumor-suppressive miR-708 and the inhibition of TNBC metastasis in vivo [264]. An alternative method was used in one study to develop multifunctional tumor-penetrating mesoporous silica nanoparticles (MSNs) for the simultaneous delivery of siRNA (siPlk1) and a tumor suppressor miRNA (miR-200c) to breast tumors. In this research, the scientists modified the nanosystem to allow deep tumor penetration by employing the photosensitizer indocyanine green (ICG) and surface conjugation of the iRGD peptide. Following short light irradiation, the iRGD-modified MSNs loaded with siPlk1 and miR-200c demonstrated enhanced delivery, cellular uptake, and tumor penetration in vitro, as well as considerable inhibition of primary cancer progression and metastasis in vivo [265].

### Extracellular vesicular (EV) based carriers

The use of extracellular vesicles in gene therapy is another delivery method. Exosomes, microvesicles, and apoptotic bodies are the three categories of EVs based on their biogenesis [266]. Extracellular vesicles, such as exosomes, are released by a variety of cell types, including tumor cells, and serve as natural carriers of miRNAs. After being absorbed by recipient cells, these miRNAs cause subsequent reactions [267]. Due to their improved biocompatibility and high delivery effectiveness, this intriguing event suggests extracellular vesicles as potential miRNA delivery vehicles for breast cancer treatment. As an example, Ohno et al. found that delivering tumor suppressor let-7a miRNA using exosomes from HEK-293 cells to EGFR-expressing xenograft breast cancer tissue in mice inhibits the growth and development of tumors [268, 34]. In another report, to deliver anti-miR-21 to 4T1 breast cancer cells, a nanoplatform based on tumor cell-derived extracellular vesicles (TEV) was created. Gold-iron oxide nanoparticles (GIONs) were then functionalized in TEVs to produce TEV-GIONs, which showed the promise of TEV-GIONs for simultaneous treatment employing miRNA and cancer imaging while enhancing tumour-specific targeting [269]. In order to study the possibility for doxorubicin and hydrophobically modified miR-159 to be delivered together for the treatment of triple-negative breast cancer, Gong et al. made use of exosomes as endogenous nanocarriers. Exosomes from differentiated THP1 monocyte cell culture-derived macrophages were loaded by doxorubicin and miR159 incubation and subsequently Exosome treatment of TNBC cells led to an enhanced anti-cancer outcome and inhibit cancer cell invasion [270]. Of note, Extracellular vesicles from primary cells provide a safer option for delivering miRNA or anti miR oligos, however it can be difficult to generate enough of these vesicles for therapeutic purposes. In order to get around these restrictions, Usman et al. recently demonstrated the utilization of extracellular vesicles produced from red blood cells to transport anti-miR-125b for a successful therapy of leukemia and breast cancer both in vitro and in vivo and results of this report indicated that treated tumors have had little invasion and metastasis [197, 271–273]. Recently, Sheykhhasan et al. successfully delivered anticancer miR-145 into breast cancer cells using exosome derived from adipose tissue-derived mesenchymal stem cells (AT-MSC-exos). Results of this research revealed that MiR-145 was transferred to BC cells by AT-MSC-exos, and this increased miR-145 level in breast cancer cells can induce apoptosis and impede breast cancer metastasis by regulating the expression of several genes such Rho-Associated Coiled-Coil Containing Protein Kinase 1 (ROCK1), Erb-B2 Receptor Tyrosine Kinase 2 (ERBB2), Tumor Protein p53 (TP53), and Matrix Metalloproteinase 9 (MMP9) [274, 275].

## CRISPR/Cas9 as a targeted therapy in breast cancer

The clustered regularly interspaced short palindromic repeat (CRISPR)-associated protein-9 (Cas9) has recently emerged as a gene-editing tool and revolutionized genome-editing approaches in a variety of biological areas, including gene therapy and human cancer research. This may be explained by its adaptable features, such as high specificity, accuracy, time and cost-saving procedures, and risk-minimized approaches. This technique can be used to target oncogenes and tumor suppressor genes to decrease tumor progression through various mechanisms such as knocking out, gene editing, repression, and epigenetic modifications [276]. In a variety of cancer models, including leukemia, cervical cancer, prostate cancer, endometrial cancer, ovarian cancer, and breast cancer, CRISPR/Cas9 has been successfully employed to eliminate both cellular and viral oncogenes (see review [277]). For instance, in a research by Schuijers et al., the MYC gene was downregulated using CRISPR/Cas9, which has been linked to a 30–50% increase in high-grade breast tumors and increased expression of the gene. As a result, c-MYC has been regarded as a primary target in the treatment of cancer [278]. In regard to migration and metastasis, in MDA-MB-231 breast cancer cells, Yang et al. used CRISPR/Cas9 technology to induce CXCR7 or CXCR4 knockout or co-knockout. According to a clinical investigation, TNBC has a poor prognosis and a greater propensity to metastasis when CXCL12 and its receptors CXCR4 and CXCR7 are expressed more. The results of this research also showed that co-knocking down CXCR4 and CXCR7 may greatly reduce TNBC, suggesting to a synergistic function for these two receptors in the development of TNBC [279]. CRISPR/Cas9 has the ability to edit or remove non-coding RNA regions. The CRISPR/Cas9-derived miR-23b and miR-27b miRNA knockout MCF-7 cell line was developed by Hannafon et al. They discovered that both of these miRNAs have the potential to cause cancer, however miR-27b may also have tumor-suppressor function in some situations [280]. In another study, it has been revealed that in vivo TNBC tumor development and metastasis are inhibited by E3-Ubiquitin Ligase (UBR5) deletion using the CRISPR/Cas9 system. UBR5 has been regarded as a driver of tumor development and metastasis in breast cancer as a result [281].

## Challenges in use of miRNA-based therapeutics

Features of RNA oligonucleotides make drug design more challenging. The following challenging traits are among them: (i) nuclease degradation after addition to biological systems [282, 283] (ii) Poor penetration of the cell membrane [284] (iii) endosomal entrappment [285] (iv) a low affinity for binding to complementary sequences [286] (v) ineffective delivery to intended target tissues [285] (vii) the induction of innate immune responses and (vi) off-target and undesired toxicities [287].

### Degradation of unmodified miRNAs

Maintaining the stability and uniformity of miRNAs in circulation is a challenge in miRNA therapies. Nucleases such serum RNase A-type nucleases in the blood quickly breakdown naked miRNAs with an unaltered 2′ OH in the ribose moiety [288]. Additionally, the fast renal excretion clearance of naked miRNAs results in a short half-life in circulatory system [289, 290]. Chemically designed miRNA alterations on the 2’ of the ribose and the phosphodiester backbone, which prevent the miRNA from degrading and increase its long-lasting potency, are the solution to this issue [288, 291]. There have been several chemical alterations created, including phosphodiester links, ribose backbone, 2'-O-(2-Methoxyethyl), 2'-O-Methyl, 2'-locked nucleic acid, and 2'- Fluoro [292].

These alterations not only increase oligonucleotide stability but also boost binding affinity to the target and aid loading into the miRNA-induced silencing complex, both of which enhance miRNA functionality [292].

### Poor miRNA penetration

The main obstacle of miRNA treatments is effective delivery to the target tissue with efficient penetration of the payload to a particular spot. We discussed the significant advances in development of the carriers in miRNA delivery in the previous section (Viral and non-viral based carriers).

### Unwanted on-target effects and Off-target effects

miRNAs when they are discharged from the endosome and transported into the cytoplasm, one of the main obstacles related to miRNA-based therapy is the off-target effect. Because incomplete hybridization with 3′ UTRs produces miRNAs that target other pathways, they may unintentionally silence other genes. Off-target gene silencing may have harmful consequences and have diminished therapeutic benefits. The likelihood of unanticipated side effects is suggested by the research showing that a single miRNA may target many mRNAs. Even if a particular miRNA is successfully targeted, there may still be unintended side effects [293]. One strategy to get around this problem is to utilize small amounts of combined miRNAs that work together to control the expression of the same target gene [294, 295].

### Activation of immune system

The host system may identify double-stranded RNAs as pathogens, and the innate immune system may identify them and become activated. For instance, systemic miRNA administration can stimulate the innate immune system, much like other forms of nucleic acids, which can have toxicities and serious adverse consequences [296]. Type I interferons (IFNs) and inflammatory cytokines can be released through Toll-like receptors in response to systemic delivery of miRNA duplexes (TLRs). Single or double-stranded RNAs (dsRNAs) can activate TLRs 3, 7, and 8 to trigger innate and adaptive immune responses. The type I interferon (IFN) pathway and cytokine production are both activated when these TLRs detect dsRNA molecules in lysosomal compartments and cellular endosomal [297]. Although research on immunological responses to miRNAs is ongoing, it is evident that the chemical alterations could decrease immune system recognition [283].

The immune system's response to the delivery vehicle's toxicity and potential to activate the immune system, which is extremely positively charged, is another intriguing aspect. Immune system interactions are unavoidable once nanoparticles reach the body. The main factors that influence how nanoparticles interact with the immune system include size, shape, surface alterations, and hydrophobicity of nanoparticles [298–302]. Therefore, it is necessary to investigate both the miRNA and the delivery method. It will be possible to provide lower doses and, afterwards, probably diminish immune reactions if the carrier is targeted to the particular tissues.

## Conclusion

Study on BCa has focused on decoding the beginning and developmental mechanism of the neoplastic modifications that arise at the epithelial tissue of the breast gland. Despite the fact that angiogenesis appears to have an important role, long term contemporary researches have revealed the dynamic participation of microRNAs in BCa. Additionally, over the last decades, microRNAs have demonstrated to be capacity dependable prognostic markers for BCa progression and universal survival, and that would also be used for tracking patient’s progression or even for the choice of the correct treatment. Within the past few a long time, the relationships between miRNAs and BCa have been broadly examined, contributing significantly to studies of breast cancer pathogenesis and their clinical suggestions. These small molecules play critical parts within the oncogenesis, development, invasion, metastasis, and angiogenesis of breast cancer; in this way, modified expression of miRNAs can be respected as a target for the conclusion and/or treatment of breast cancer based on the miRNA expression profile. However, numerous of the remaining issues critically require advance investigation. For illustration, we know little about the upstream, natural variables that control these particular miRNAs; there are tens or hundreds of miRNAs inside a cell, and which is the foremost noticeable marker for the determination and treatment as well as estimating of cancer remains to be illustrated. Future research should focus on identifying the limited miRNAs implicated in angiogenesis of breast cancer, and also therapeutic targets. To date, there has been different research and considerable breakthroughs in understanding the processes and efficiency of miRNA therapies, but particular challenges to maximum efficiency are still unanswered. Stability, specificity, targeted delivery, toxicity, and immunological activation are some of these difficulties. Despite these encouraging developments, miRNA therapeutic candidates have yet to enter phase III clinical trials and acquire FDA approval for medical intervention. The effective translation of miRNA-based strategies from bench to bedside is dependent on the advancement of miRNA delivery vehicles that combine crucial features such as stability, high loading capacity, increased half-life in circulation, prevention of cargo degradation, and minimal toxicity. The inventory of new miRNAs related with human health and illness will continue to grow over the next decade, thanks to recent breakthroughs in next-generation sequencing (NGS) methods and bioinformatic tools. A potential research field will be the development of novel delivery systems and their testing in animal models. Furthermore, future research should concentrate on the identification of disease-specific markers on target tissues as well as the development of novel targeting ligands to improve miRNA therapeutic effectiveness.

## Data Availability

Not applicable.

## Abbreviations

Pri-miRNA: Primary microRNA
Pre-miRNA: Precursor microRNA
AGO2: Argonaute protein fadown-regmily
RISC: RNA induced silencing complex
