# Correction: The various role of microRNAs in breast cancer angiogenesis, with a special focus on novel miRNA-based delivery strategies

**DOI:** 10.1186/s12935-023-02885-y

**Published:** 2023-03-25

**Authors:** Min Yang, Ying Zhang, Min Li, Xinglong Liu, Mohammad Darvishi

**Affiliations:** 1 College of Traditional Chinese Medicine, Jilin Agricultural Science and Technology University, Jilin, 132101 China; 2 Infectious Diseases and Tropical Medicine Research Center (IDTMRC), Department of Aerospace and Subaquatic Medicine, AJA University of Medical Sciences, Tehran, Iran

**Correction: Cancer Cell International (2023) 23:24** **https://doi.org/10.1186/s12935-022-02837-y**

In this article [1], the grant number was not stated. The acknowledgment to be amended as follows:

- The research is supported by the Science and Technology Research Project from the Education Department of Jilin Province, Based on the NF-κB mediated inflammatory corpuscle and PI3K/AKT signaling pathway research puerarin drug mechanism of nanometer fiber membrane on the skin wound healing (No. JJKH20210413KJ).
